# Supplementary material for: Is Hemopexin a Nephrotoxin or a Marker of Kidney Injury in Renal Ischemia-Reperfusion?
Source: Biomolecules. 2024 Nov 27;14(12):1522. doi: 10.3390/biom14121522 (PMC11673696; doi:10.3390/biom14121522)
Supplement: Supplementary file 1 [file biomolecules-14-01522-s001.zip › biomolecules-3331813-supplementary.pdf]

Supplementary Materials: Figure S1. Raw data for Western blots in Figure 2.

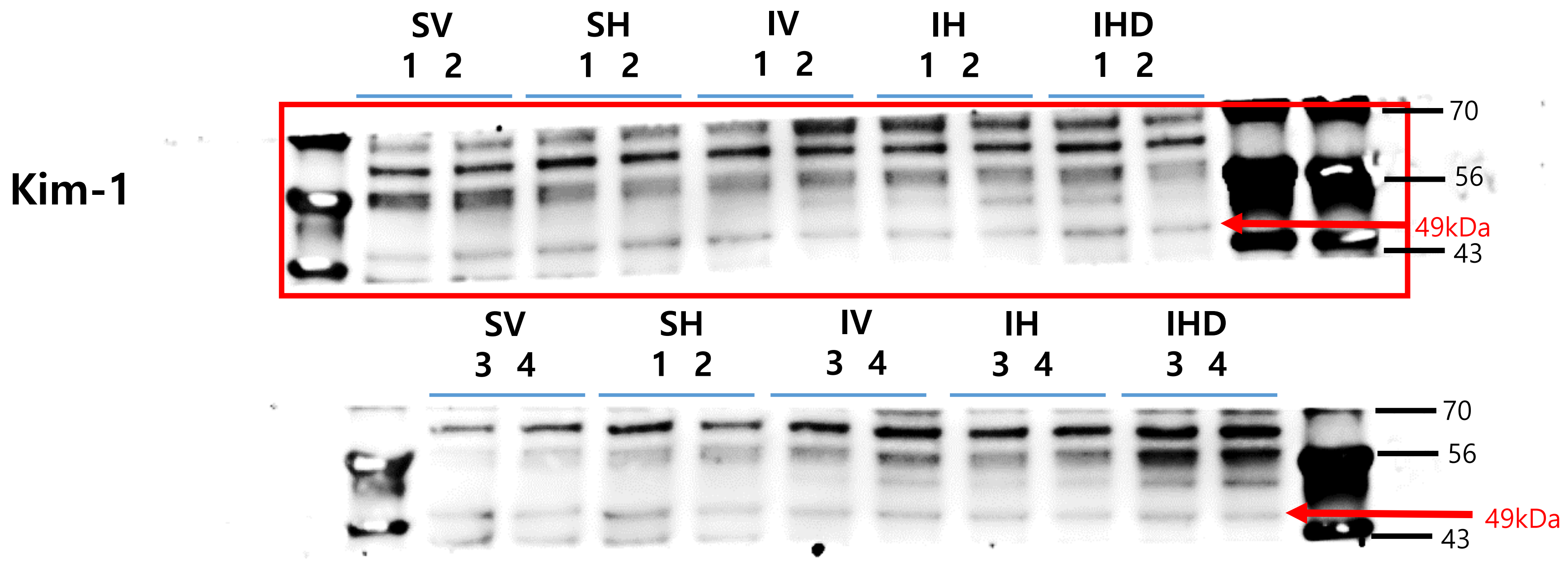

NGAL

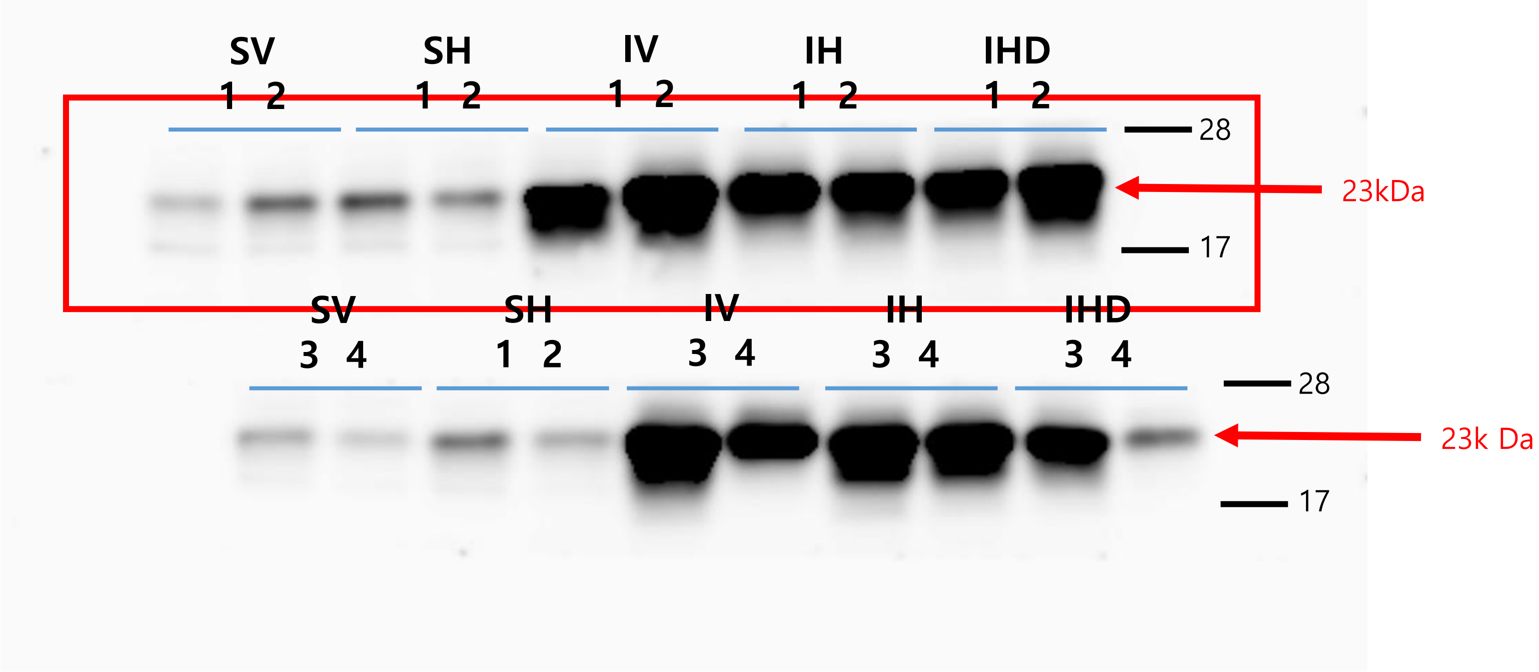

SV
1 2
SH
1 2
IV
1 2
IH
1 2
IHD
1 2
28
23kDa
17
SV
3 4
SH
1 2
IV
3 4
IH
3 4
IHD
3 4
28
23k Da
17

Fibronectin

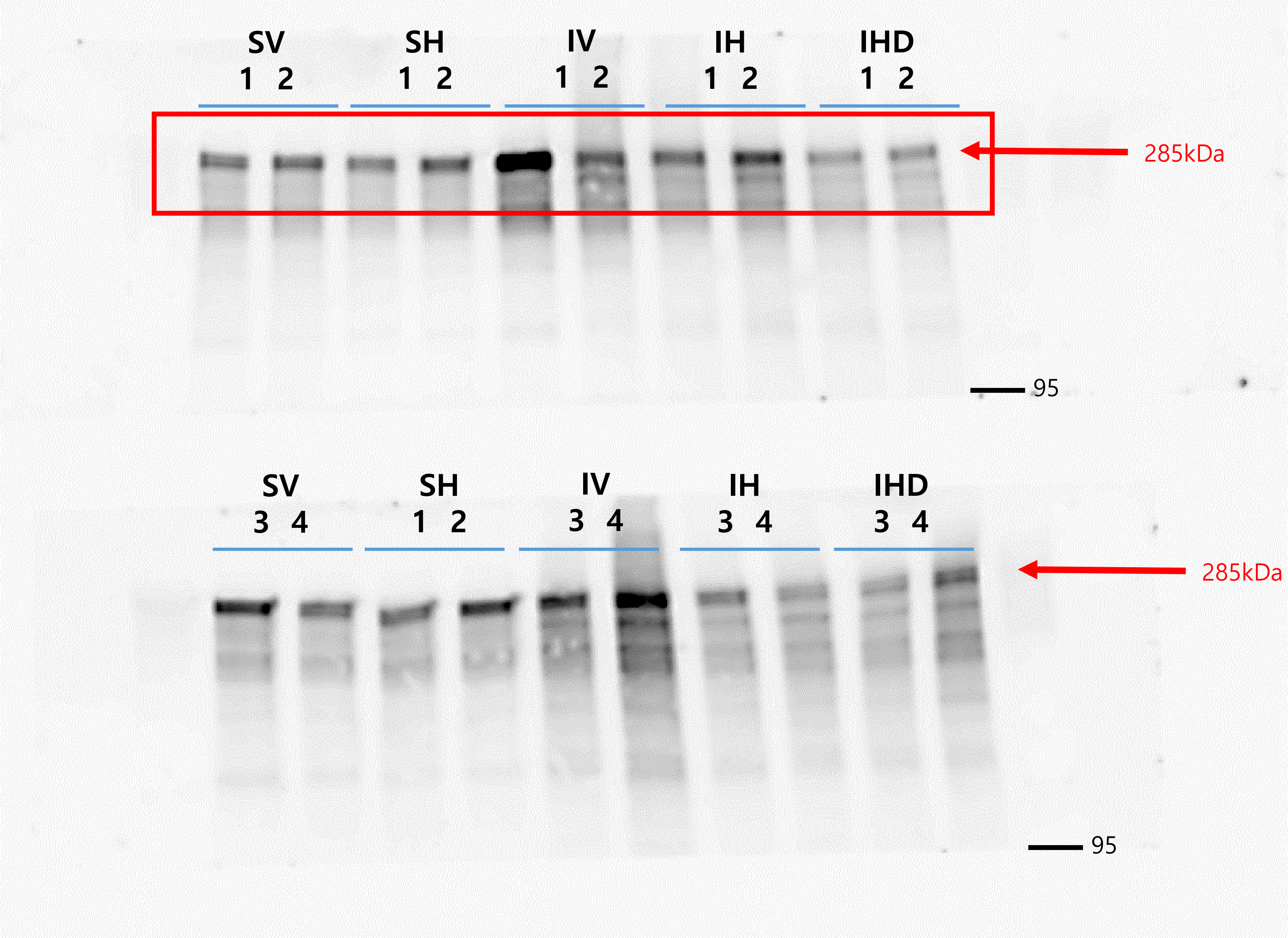

HO-1

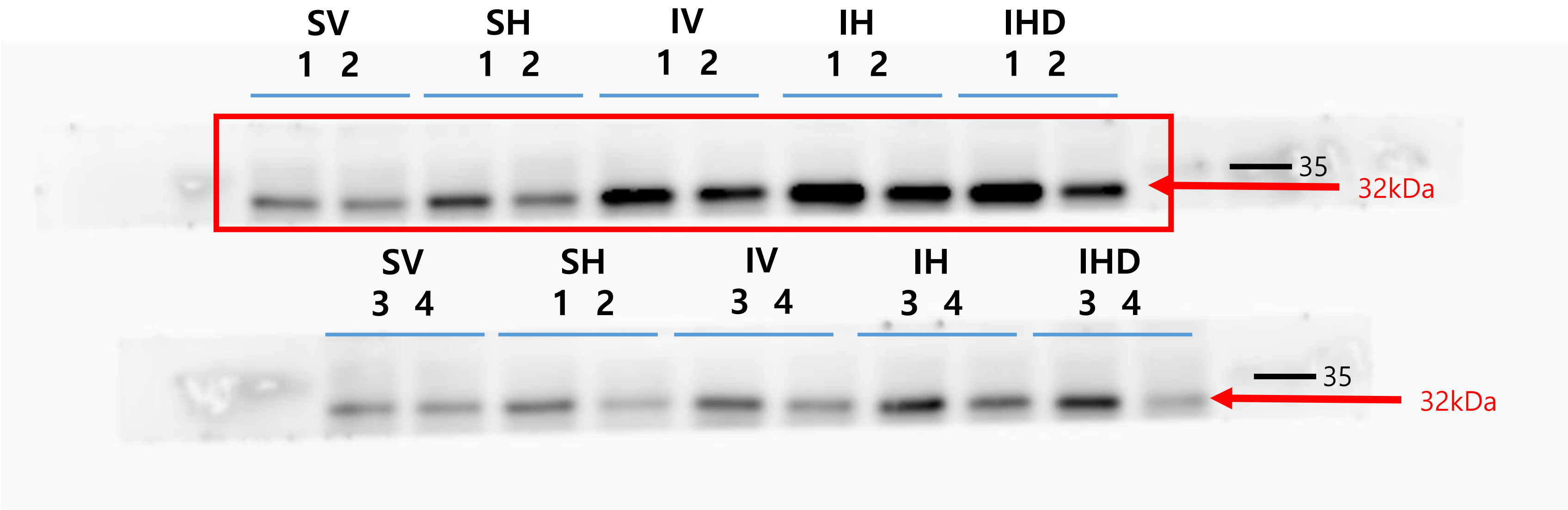

SV
1 2
SH
1 2
IV
1 2
IH
1 2
IHD
1 2
35
32kDa
SV
3 4
SH
1 2
IV
3 4
IH
3 4
IHD
3 4
35
32kDa

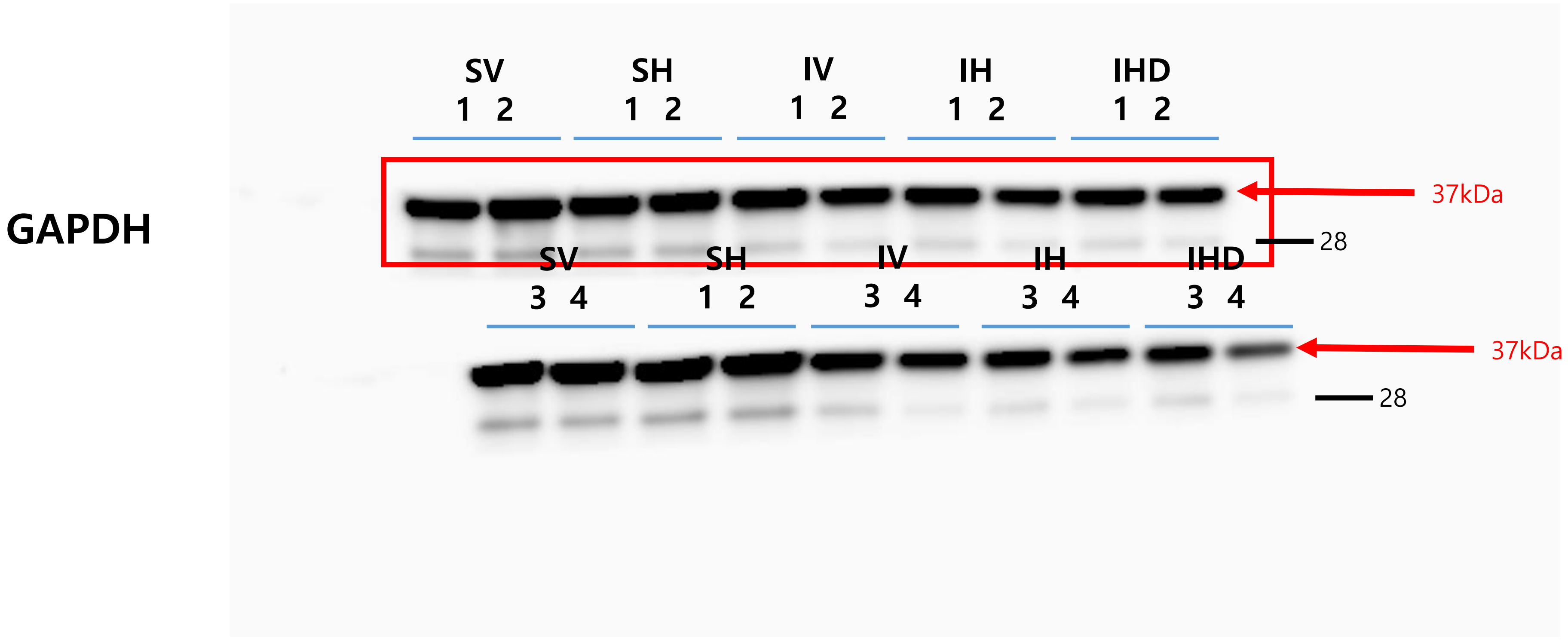
GAPDH
SV
1 2
SH
1 2
IV
1 2
IH
1 2
IHD
1 2
37kDa
28
SV
3 4
SH
1 2
IV
3 4
IH
3 4
IHD
3 4
37kDa
28

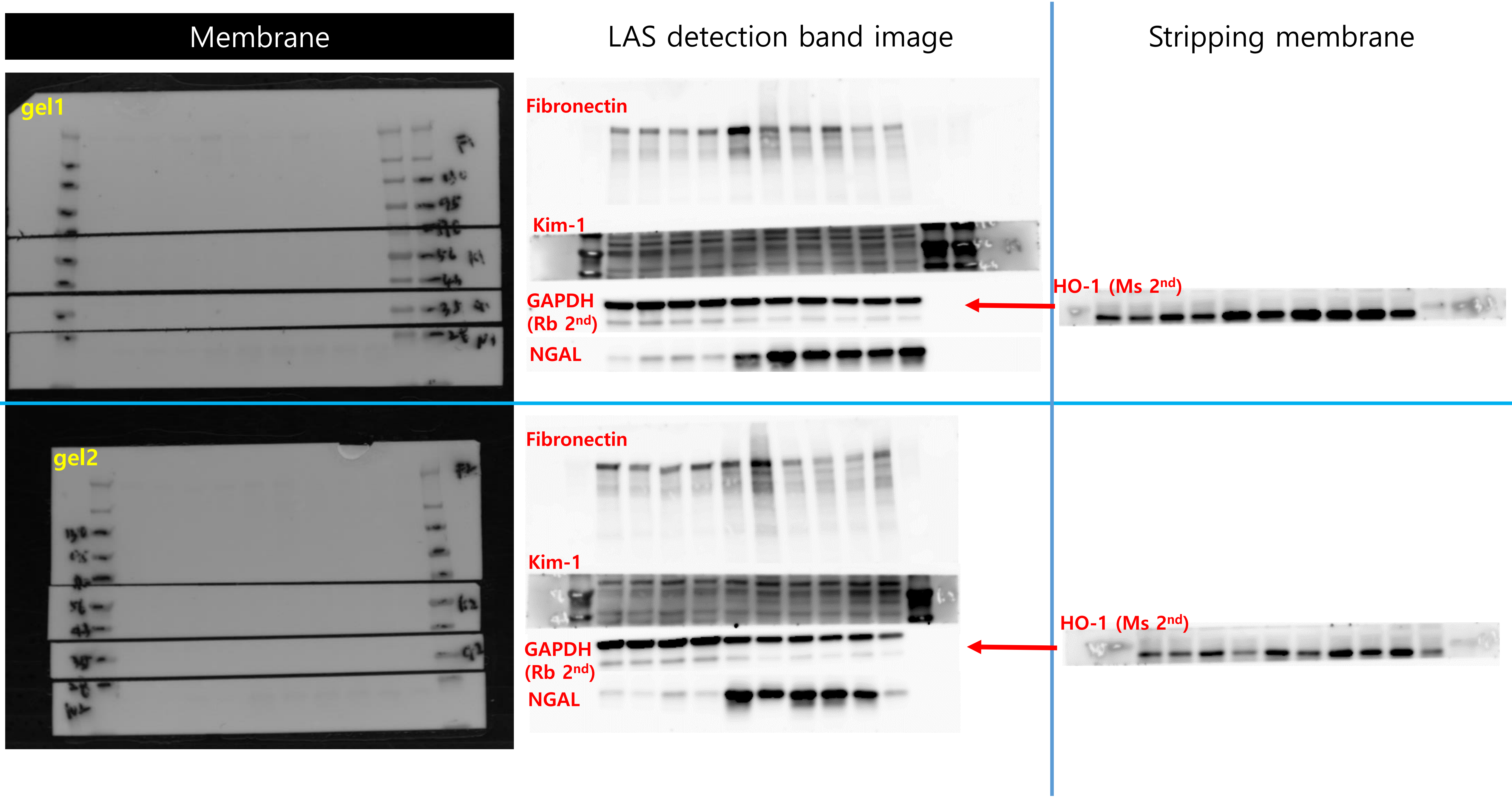
Membrane
LAS detection band image
Stripping membrane
gel1
Fibronectin
Kim-1
GAPDH
(Rb 2nd)
NGAL
HO-1 (Ms 2nd)
gel2
Fibronectin
Kim-1
GAPDH
(Rb 2nd)
NGAL
HO-1 (Ms 2nd)
